# Handlebar Injuries: Not Always the Pancreas

**DOI:** 10.7759/cureus.42560

**Published:** 2023-07-27

**Authors:** Christopher J Cacciatore, Katharina Pellegrin, David Kashmer, Nathan P Holtman

**Affiliations:** 1 Surgery, Edward Via College of Osteopathic Medicine, Auburn, USA; 2 Surgery, Naval Medical Center, Portsmouth, USA; 3 Surgery and Simulation, Edward Via College of Osteopathic Medicine, Auburn, USA

**Keywords:** pediatric trauma, motorcycle handle, blunt abdominal injury, emergency medicine and trauma, handlebar injury, fast exam, general surgery, gall bladder trauma, motor vehicle collison, general trauma surgery

## Abstract

This case report highlights a rare traumatic gallbladder rupture secondary to a handlebar impact to the abdomen. Traumatic gallbladder rupture is only seen in 1.9-2.1% of all reported abdominal trauma. The diagnosis can be delayed due to the rarity of injury and the non-specific symptoms that a patient may present with. This case highlights the need for high clinical suspicion based on the mechanism of injury and imaging studies (focused assessment with sonography (FAST) and computed tomography (CT) scan) to direct treatment of concurrent injuries to assure the best outcome and prevent complications and morbidity. This patient was treated surgically with cholecystectomy and was discharged in stable condition.

## Introduction

Direct handlebar impact is most likely to cause injuries to the pancreas, small bowel, mesentery, liver, and spleen [1]. In the pediatric population, the relatively thin body wall may increase the risk of abdominal injury following the impact. On the other hand, traumatic gallbladder rupture is extremely rare, occurring in 1.9-2.1% of all reported abdominal trauma [2]. Traumatic gallbladder rupture comes with some serious complications, such as bile leak, infection, necrosis, and even hemobilia. Factors that are hypothesized to contribute to this rarity include the gallbladder’s protected anatomical location, decreasing number of children engaging in outdoor activities, and the smaller impact target offered by the gallbladder [1].

We are reporting a case of a 17-year-old male who presented to an urgent care facility in moderate distress with upper abdominal pain and episodes of emesis after he collided with his motorcycle handlebars. His mechanism of injury and primary survey demanded rapid computed tomography (CT) imaging of the abdomen, which demonstrated a gallbladder laceration extending deep into the liver parenchyma. This case magnifies the importance of the expeditious and accurate workup of minimally symptomatic blunt trauma patients, as they may still have significant injuries requiring emergent interventions.

## Case presentation

A 17-year-old male patient was brought into an urgent care facility by his mother hours after sustaining a blunt force trauma to the abdomen. He was traveling at an unknown speed toward another motorist when he quickly shifted direction and impacted the handlebar in his right upper quadrant (RUQ). The patient initially presented to an outside hospital, where he received an initial workup. Subsequently, he was transferred to our hospital for further evaluation. On physical examination, there were no signs of superficial ecchymosis, but a slender boy in moderate distress complained of abdominal pain and reported several episodes of emesis. He denied any loss of consciousness or peri-event amnesia. Due to the mechanism of injury, which raised concern for abdominal injury, the patient was taken for a CT scan in stable condition.

**Figure 1:**
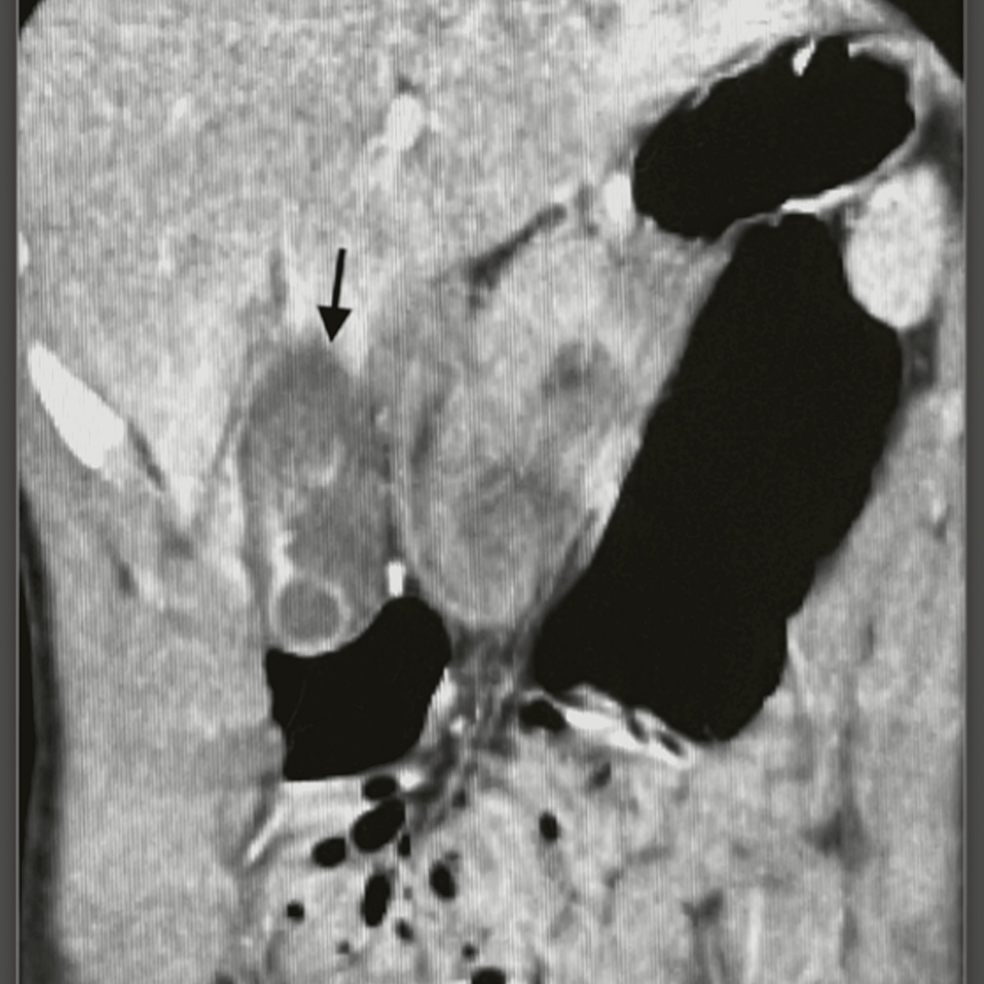
Black arrow showing the pericholecystic fluid accumulation

**Figure 2:**
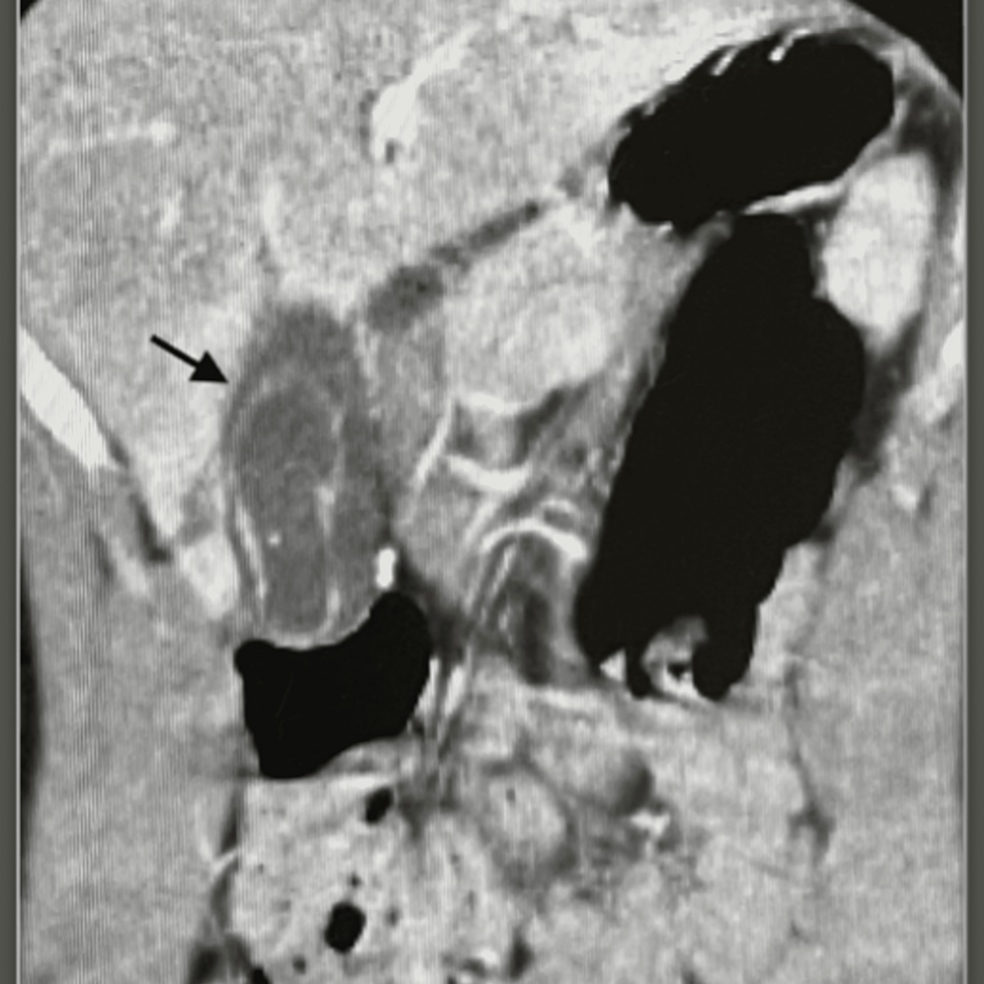
Black arrow showing the pericholecystic fluid accumulation

**Figure 3:**
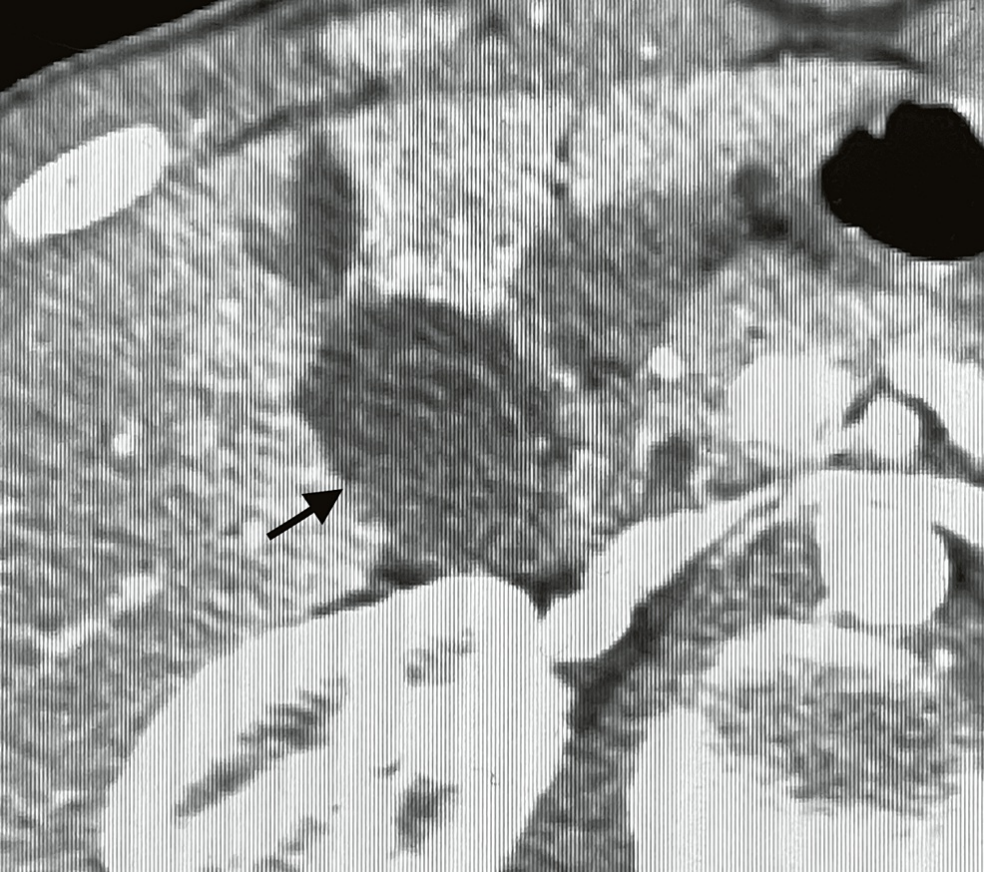
Black arrow showing the pericholecystic fluid accumulation

CT imaging showed contrast extravasation within the gallbladder as well as rupture of the gallbladder wall with surrounding hematoma, which is classified as a grade 2 gallbladder injury according to Losanoff and Kjossev, as shown in Table 1 [3]. The CT demonstrated an American Association for the Surgery of Trauma (AAST) grade II liver laceration (1.2 cm in length). As shown in Figures 1-3, the only injuries appreciated on CT for our patient were to the gallbladder and liver, which showed pericholecystic fluid accumulation and a small liver laceration. 

**Table 1:** Classification of gallbladder injuries Proposed by Losanoff and Kjossev [3]

Type	Description	Proposed treatment
1A	Contusion + intramural hematoma	Conservative/cholecystectomy
1B	Contusion + intramural hematoma + necrosis + eventual perforation	Cholecystectomy
2	Wall rupture at injury	Cholecystectomy
3A	Partial avulsion	Conservative/cholecystopexy/cholecystectomy
3B	Complete avulsion with hepatoduodenal ligament intact	Cholecystectomy
3C	Hepatoduodenal ligament detached: liver bed intact	Cholecystectomy
3D	Total avulsion/traumatic cholecystectomy	Hemostasis/cystic duct clip
4A	Traumatic cholecystitis	Cholecystectomy + evacuation of hemobilia
4B	Acalculous cholecystitis complicating trauma	Conservative/cholecystectomy
5	Mucosal tear (gallbladder wall intact)	Cholecystorraphy/cholecystectomy

 

 

The patient was then moved expeditiously to the operating room. Three hours post-injury, exploratory laparotomy through a midline incision confirmed a segment IV liver laceration with active bleeding that was rapidly controlled with cautery. There was no evidence of bile in the peritoneal cavity, and a subserosal hematoma throughout the gallbladder wall was identified. This was addressed with an open cholecystectomy with irrigation of the RUQ. Next, the liver laceration was more definitively addressed with a hepatorrhaphy using chromic sutures. Pathology report of the gallbladder specimen noted a 6 × 4 × 4 cm (0.2-0.3 cm in thickness) gallbladder with hemorrhage subserosally. 

His postoperative course was uneventful, and he was discharged home on postoperative day four.

## Discussion

The evaluation of blunt trauma patients has significantly changed over the past 30 years. The new trauma protocols emphasize expeditious initial assessment to determine the next step with further imaging or intervention (operating room/interventional radiology). The spleen and liver are the most commonly injured solid organs in blunt abdominal trauma (BAT), while the small bowel is the most commonly injured hollow viscus in BAT [2]. Traumatic gallbladder rupture is an infrequent occurrence seen only in 1.9-2.1% of all abdominal trauma [2]. 

The low incidence of acute gallbladder rupture may be related to a variety of factors. The relatively small size of the gallbladder (average 9 cm × 4 cm) provides some inherent protection against injury. Moreover, the proximity of the gallbladder to the liver and neighboring abdominal organs, such as the stomach, intestines, and omentum, offers additional shielding [4]. These neighboring structures likely absorb and dissipate some of the impact force during trauma, reducing the likelihood of gallbladder injuries.

Due to the proximity of the liver, a gallbladder injury should be suspected, with BAT impacting the RUQ. We believe that a shearing force across the RUQ with a direct blow to the abdomen contributes to the vulnerability of the gallbladder. As in this case, there was a handlebar impact to the abdomen, most likely with a rotational component, which made the gallbladder susceptible to injury.

There are other important factors to consider aside from the mechanism of injury predisposing to laceration of the gallbladder. A distended, thin-walled gallbladder puts patients at a greater risk for rupture if involved in a traumatic injury to the abdomen. If the patient was in a postprandial state, it may put them at an increased risk due to further distension. Distention of the gallbladder occurs in a postprandial state due to the production of bile in preparation for the consumption of a patient’s next meal. The bile residing in the gallbladder places additional strain on the wall, which can lead to an increased chance of rupture if BAT is localized to the RUQ. Alcohol consumption results in an increase in the tone of the sphincter of Oddi with a consequent increase in the biliary tract pressure and distention of the gallbladder [5]. Either the increased biliary tract pressure from a postprandial state or alcohol consumption can each, respectively, increase the risk of gallbladder rupture and, if it happens concurrently, can have synergistic effects, causing the patient to have a highly friable gallbladder. Intriguingly, a diseased gallbladder, characterized by fibrosis and a thickened wall, appears to have protection against rupture. The fibrosis may contribute to its increased structural support, which consequently decreases the susceptibility to rupture [3].

The diagnosis of gallbladder rupture can be extremely difficult in the acute stage before imaging. In one review of 22 gallbladder ruptures, the average onset of symptoms (i.e., nausea, vomiting, and abdominal pain) ranged from three to seven days [6]. This highlights the necessity for a close diagnostic protocol to avoid possible complications of blood loss and infection. Clinical findings vary greatly with BAT injuries. Some common findings associated with intra-abdominal trauma are seatbelt signs, rebound tenderness, abdominal distension/guarding, abdominal wall bruising, perineal hematoma, and hemodynamic instability. Distracting injuries (i.e., femur fracture) may impact the ability to obtain a reliable physical exam [6].

Due to the lack of specific signs on initial presentation and the paucity of defining symptoms indicative of a gallbladder rupture, advanced imaging studies are paramount for diagnosis. The focused assessment with sonography (FAST) exam is now routinely done on trauma patients upon arrival to the trauma bay. In some cases, visualization of specific ultrasound findings, such as hyperdense fluid with a distended gallbladder, may provide valuable clues that point to a potential gallbladder rupture [5]. However, it is essential to emphasize that interpreting these ultrasound findings accurately requires advanced sonographic skills, which are typically possessed only by a select few experienced trauma surgeons.

A CT scan should therefore be performed in a hemodynamically stable patient to further evaluate suspicious sonographic findings or if a FAST exam was not conducted. CT findings such as mural wall thickening, pericholecystic fluid and/or mass effect on the surrounding organs, the imprecise contour of the gallbladder, discontinuity of the gallbladder wall, active arterial intraluminal extravasation, and presence of a site of wall disruption or non-enhancement should raise the suspicion of a gallbladder injury [5]. Contrast extravasation may result from shearing forces that disrupt the vascular supply [5]. In our case, pericholecystic fluid was seen on CT; with the given BAT history, other causes of fluid accumulation may be ruled out.

Due to the mechanism of injury and findings on imaging, a cholecystectomy with the repair of the underlying deep liver laceration was performed in this patient. Although there was no bile visualized during the operation, a cholecystectomy is often the standard treatment for trauma to the biliary tree. According to Losanoff and Kjossev’s classification (Table 1), cholecystectomy is often preferred in many types of gallbladder injuries. Only for type 1A injuries, gallbladder contusion with an intramural hematoma, is conservative treatment recommended. In specific cases with isolated injuries to the gallbladder, some reports have demonstrated endoscopic retrograde cholangiopancreatography (ERCP) as an intervention [7]. 

Furthermore, a gallbladder rupture can present with minimal symptoms or even remain asymptomatic, posing challenges for timely diagnosis. Delayed surgical treatment in such cases may lead to increased risks of complications and adverse outcomes. This case report serves as a crucial reminder of the necessity for rapid imaging in patients with BAT and handlebar injuries, even in the presence of minimal symptoms. Early detection and intervention are vital to prevent potential complications and improve patient outcomes.

## Conclusions

Traumatic gallbladder rupture following BAT is an infrequent occurrence with potentially serious consequences such as bile leak, infection, necrosis, and even hemobilia. The anatomical protection and small size of the gallbladder often spare it from injury. However, direct impact to the RUQ, alcohol consumption, and a postprandial state increase the susceptibility of it to rupture. Diagnosing gallbladder rupture in the acute stage can be difficult because of the lack of specific signs on initial presentation. Thus, an expeditious workup, including advanced imaging studies such as CT and FAST exam, is critical for early and accurate diagnosis. Surgical exploration with cholecystectomy and treatment of concomitant injuries ensures the best outcome and minimizes complications and morbidity. This case report emphasizes the importance of maintaining a high clinical suspicion in patients with a history of BAT, particularly from handlebar impact, to rapidly screen for possible gallbladder rupture. Prompt identification and intervention are crucial for optimizing patient outcomes and preventing adverse complications.​​​​​
